# A Longitudinal Nationwide Study of Psychological Distress During the COVID-19 Pandemic in Chile

**DOI:** 10.3389/fpsyt.2022.744204

**Published:** 2022-02-24

**Authors:** Fabián Duarte, Álvaro Jiménez-Molina

**Affiliations:** ^1^Department of Economics, Faculty of Economics and Business, Universidad de Chile, Santiago, Chile; ^2^Millennium Nucleus in Social Development (DESOC), Santiago, Chile; ^3^Millennium Nucleus to Improve the Mental Health of Adolescents and Youths (Imhay), Santiago, Chile; ^4^Millennium Institute for Research in Depression and Personality (MIDAP), Santiago, Chile; ^5^Faculty of Psychology, Universidad Diego Portales, Santiago, Chile

**Keywords:** COVID-19, lockdown, social determinants of mental health, psychological distress, anxiety symptom, depressive symptom, longitudinal survey, Chile

## Abstract

**Background:**

Despite numerous efforts to assess the impact of the COVID-19 pandemic on mental health, there are few longitudinal studies that examine the change in the burden of psychological distress over time and its associated factors, especially in developing countries.

**Objective:**

The primary aim of this study was to assess the levels of psychological distress at two time points during the COVID-19 outbreak based on a representative community sample in Chile. The secondary aim was to identify groups that are more vulnerable to psychological distress during the pandemic.

**Methods:**

A nationally representative, longitudinal telephone survey of Chilean adults was conducted. This study analyses panel data from two waves in 2020: May 30 to June 10 and September 15 to October 9. A total of 823 people participated in both surveys. Changes in mental health outcomes (anxiety and depressive symptoms) were assessed, estimating the effect of demographic characteristics, psychosocial and economic factors, household conditions, and health status.

**Results:**

There was a significant increase in psychological distress (PHQ-4 ≥ 6) between Waves 1 (22.6%) and 2 (27.0%), especially among younger participants. Overall, the results of this study show that being female, living in or near the capital, living in overcrowded households and having a perceived lack of space in the home, loneliness or perceived social isolation, and having received mental health treatment within the last year are significantly associated with psychological distress over time (*p* < 0.05).

**Conclusion:**

This study highlights the need to implement psychosocial programs to protect people's psychological well-being, as well as social policies to improve household living conditions and levels of social connectedness during the COVID-19 outbreak.

## Introduction

Several studies have suggested that the disruption to social life derived from COVID-19 pandemic has imposed a huge mental health burden on society (1, 2). However, most of these studies have used cross-sectional designs and are based on convenience samples (1, 3).

Current longitudinal evidence based on community samples suggests that high levels of psychological distress were most frequently observed during the first months of the pandemic (1). These levels of psychological distress were higher than they were prior to the outbreak (4–6). New research suggests that there has been no change or even a decrease in the prevalence of psychological distress in general populations during those first few months (7–11).

However, the pandemic does not affect the mental health of different population subgroups in the same way. Several risk factors for anxious and depressive symptoms during the COVID-19 pandemic have been identified in the emerging literature, including being female (12, 13), younger than 40 years old (2, 4), preexisting physical and mental health conditions (2, 14), lower household incomes, less education, lower social support, and higher perceived loneliness (12, 13, 15). Other risk factors include living in urban areas and small households (1, 16), prolonged quarantine periods (1), and children in the household (1, 9). Food and economic insecurity related to increased unemployment and lost income have also been shown to be important risk factors for psychological distress during the pandemic (17, 18). Thus, the negative impact of the pandemic on mental health differs by demographic characteristics, health and economic status, housing conditions, and social resources.

To date, most studies regarding mental health and COVID-19 pandemic have been conducted in high-income countries and in Asia (1). However, it has been suggested that the impact of COVID-19 could be worse in developing regions, and in states with weak social safety nets (19, 20). For example, despite the implementation of strict mitigation measures, the pre-pandemic conditions that characterize Latin American countries (high poverty and inequality, informal employment, and vulnerable populations) have undermined the effectiveness of state responses to the pandemic (21).

Chile is a mid-to-high-income country characterized by comparatively high income inequality (22), poor housing conditions for many (23), and a relatively high mental illness burden with low access to mental health services (24). Since mid-March, the Chilean government has introduced strict physical distancing measures and severe restrictions on movement. Since then, many Chileans have faced serious economic difficulties (21). By July-August 2020, the national unemployment rate was 13.5% (25), the highest in the past decade. For several months in 2020, Chile had a combination of high COVID-19 incidence and mortality with prolonged periods of quarantine (21), which could have a significant impact on mental health. A study conducted in May-June 2020 found a 19.2% point prevalence of moderate to severe anxious and depressive symptoms in a representative sample of the adult population (26). This study suggested that being female, perceived loneliness, having a previous mental health diagnosis, and the expectation of reduced income due to less work because of the pandemic were significantly related to psychological distress.

Despite efforts to assess the impact of the pandemic on mental health in Chile, there is no longitudinal nationwide study estimating changes in the prevalence of psychological distress over time and its associated factors. To address this gap, the primary aim of this study was to assess the levels of psychological distress at two time points during the COVID-19 outbreak based on a representative sample of the adult population. The secondary aim was to identify the groups that are the most vulnerable to psychological distress during the pandemic. Based on the emerging COVID-19 literature, we had expected a slight decrease in psychological distress between Wave 1 (May–June 2020) and Wave 2 (September–October 2020) due to the reduction in the pandemic's severity, the relaxation of quarantine and physical distancing measures, a likely decrease in economic uncertainty, and adapting to living with pandemic-related restrictions.

## Materials and Methods

### Procedure

Social Thermometer is a longitudinal, representative, telephone survey of the Chilean adult population including urban and rural areas across the country. This study uses data from two time points. The first wave (W1) data were collected from May 30 to June 10, 2020 (*n* = 1,078). W1 coincides with one of the most critical periods of the COVID-19 outbreak in Chile and the implementation of the most severe physical distancing, including a massive lockdown (21). The second wave (W2) data were collected from September 15 to October 9, 2020 (*n* = 823). W2 coincides with a decrease in the pandemic's severity, relaxation of quarantine measures, and the progressive return to normal social lives.

The construction of the sample was multi-stage. The first stage emerged from a probabilistic and geographically stratified sampling strategy that randomly selects municipalities, then census blocks and occupied dwellings, and finally people 18 years old or older. In this process, the expansion factor was calculated as the inverse of the selection probability. It also includes adjustments for non-response and post-stratification age range adjustments (18–35, 36–59, ≥60) for men and women, in two stages. Both corrections are made to reduce telephone survey bias in accordance with ECLAC (27) recommendations.

During W1, the contact rate was 39.9% with a cooperation rate of 60.7%; the response rate was 24.2%. During W2, the contact rate was 87.8% with a cooperation rate of 87.4%, so the retention rate was 76.8%. With these values and assuming simple random sampling, and for a proportion of 0.5, the survey is nationally representative (with an absolute error of 3.4%) for men and women (with absolute errors of 4.9 and 4.7%, respectively) and for age groups 18–35, 36–59, and ≥60 (with absolute errors of 6.7, 5.1, and 6.4% respectively).

### Participants

The final sample was restricted to individuals with repeated measures for the variables of interest (*n* = 766). We ran regressions on the entire sample in each wave to check for robustness, but we decided to analyze the results for participants in both waves to reduce possible bias.

Table 1 describes the socio-demographic characteristics of the panel sample (W1–W2).

**Table 1:** Sociodemographic characteristics of the panel sample (W1–W2).

**Variable**	***n* expanded (%)**
**Sex**	
Male	10,614,636 (50.1)
Female	10,584,580 (49.9)
**Age**	
18–35	7,593,809 (35.8)
36–59	9,192,226 (43.7)
≥60	4,413,181 (20.8)
**Household income per month (USD)**	1,162 (1,172)^*^
**Education**	
Primary school (≤8 years)	1,983,305 (9.4)
High school (between 9 and ≤12 years)	8,765,916 (41.3)
Higher education (>13 years)	10,449,995 (49.3)
**Household conditions**	
Number of rooms per person	0.95 (0.53)^*^
Children at home (1 or more)	0.31 (0.46)^*^
**Geographic area (place of residence)**	
Northern Chile	1,600,517 (7.6)
Central Chile	15,194,012 (71.7)
Southern Chile	4,404,688 (20.8)

* *Mean. US$1=CLP$740*.

### Measures

We use measures identified in the COVID-19 literature for the main risk factors for psychological distress: demographic characteristics (such as gender or age), psychosocial and economic factors, household living conditions, and health status.

#### Psychological Distress

We use the Patient Health Questionnaire-4 (PHQ-4) to measure psychological distress (28). We have chosen this scale to measure psychological distress because it is an ultra-brief version of the PHQ-9 (depressive symptoms) and GAD-7 (anxious symptoms) scales, which have been used in several mental health studies during the COVID-19 pandemic (1, 2). We have chosen psychological distress (anxious and depressive symptoms) as the main outcome because it reflects one of the most common mental health problems in the general population, and because it is a dimension that is sensitive to people's socio-environmental conditions.

PHQ-4 is an ultra-brief screening tool (4-item self-report questionnaire) used for the evaluation of anxiety and depressive symptoms. The PHQ-4 is composed of a 4-point ordinal response scale (from 0 = not at all to 3 = nearly every day). A total score ≥ 6 indicates the presence of moderate to severe anxiety-depression symptoms. Although it is not a diagnostic instrument, it has demonstrated good accuracy for detecting both anxiety and depressive disorders (28). Likewise, a recent meta-analysis shows that the sensitivity of the PHQ-2 to assess depressive symptoms is higher than that of semi-structured interviews (29). In this panel sample, the PHQ-4 had an internal consistency of 0.78. Since psychological distress exists along a continuum ranging from mild, time-limited distress to severe mental health conditions, the dependent variables were regarded as continuous.

#### Mental Health Diagnosis and Treatment

We get a mental health history (“At some point in your life, have you been diagnosed with an illness such as depression, anxiety disorder, bipolar disorder, or another mental health problem?” [Yes/No]). We also evaluate access to mental health treatment during the past twelve months (“During the last 12 months, have you been in treatment for any mental health problems? (with general practitioner, psychologist or psychiatrist)” [Yes/No]). W1 responses for both were imputed to W2.

#### Physical Health Status

We measure physical health conditions through the following question: “Are you currently diagnosed with any of the following diseases or health conditions? [Hypertension, Obesity, Diabetes]” [Yes/No]. W1 responses were imputed to W2.

#### Loneliness

We used an adapted version of the Three-Item Loneliness Scale (30). It measures three aspects of loneliness: social, relational, and self-perceived connectedness. The three were merged into one question: “Over the last 2 weeks, how often did you feel that you lacked companionship, that you were being left out, or that you were isolated from others?” (0 = not at all to 3 = nearly every day). Due to the large number of survey items and the need to maximize time in a telephone survey, we decided to combine the three aspects of loneliness into a single item.

#### Household Overcrowding

Our indicator was defined as the number of bedrooms (used exclusively for sleeping) per person.

#### Perceived Lack of Space at Home

Participants were asked “In case you live in a sector that is or has been under mandatory quarantine, which of the following difficulties do you think you or other members of your household faced since the beginning of quarantine?: A lack of space at home” [Yes/No].

#### Children

Participants were asked “How many children under the age of 10 live in your household?” The scores were categorized into two groups [0 = no children; 1 = one or more children].

#### Economic Uncertainty

Defined as the expectation of facing adverse economic situations over the next 3 months, related to two questions: household income and debt. “Do you think your household's income (debt) situation over the next 3 months will be…? [1 = Better, 2 = Same, 3 = Worse]. Expectation of reduced household income and increased household debt were defined as separate indicators.

#### Basic Supplies

Participants were asked “In the event that your home is in a lockdown area, which of the following difficulties do you think you or other members of your household will have to face?: Difficulty in accessing food and basic supplies” [Yes/No].

Finally, demographic, and socio-economic variables were included. They were used in the model as continuous (age and log of income) and dummy variables (gender, geographic area, education).

### Data Analysis

First, the proportion of participants scoring above the clinical cut-off (PHQ-4 ≥ 6) and the distribution of different social, psychological, and health variables was calculated for each wave. *T*-tests were performed to estimate differences between the two waves.

Second, ordinary least-squares regression models were conducted, first as separate samples (regressions for each wave), then using the complete panel (both waves), and time fixed effects. In regression models, we considered depressive symptoms (0–6 points), anxiety symptoms (0–6 points), and psychological distress (depressive + anxiety symptoms, 0–12 points) as separate dependent variables.

Finally, we analyzed the possible differences by sex and age (18–35, 36–59, ≥60).

Expansion factors were included in the regression models' calculations. The expansion factors considered different components, including the inverse of the selection probability, corrections for non-response (by telephone), and post-stratification adjustments. Regarding non-response correction, a propensity score was estimated, using the probability of responding given certain individual observables. Post-stratification adjustments were included to reach population values by sex and age group. Following technical recommendations in the literature, we used the same expansion factors for both waves (31).

## Results

As can be seen in Table 2, our sample exhibited relatively high levels of psychological distress (PHQ-4 ≥ 6), with ~22.6–27.0% of participants reporting moderate to severe anxiety and depressive symptoms in W1 and W2, respectively. This represents a significant increase in psychological distress between W1 and W2 (*p* < 0.01).

**Table 2:** Psychosocial and health variables by study wave (*n* = 10,599,608).

**Variable**	**W1 % (95% CI)**	**W2 % (95% CI)**	**Dif**	***t*-test**	***p***
Psychological distress	22.6 (22.6–22.6)	27.0 (26.9–27.0)	4.4	236.2	0.000
Mental health diagnosis	26.0 (25.9–26.0)	–	–	–	–
Mental health treatment	15.2 (15.2–15.2)	–	–	–	–
Physical health status (hypertension, obesity and/or diabetes)	34.5 (34.4–34.5)	–	–	–	–
Loneliness	15.2 (15.1–15.2)	19.6 (19.6–19.6)	4.4	270.2	0.000
Perceived lack of space in the home	12.8 (12.7–12.8)	21.7 (21.6–21.7)	8.9	543.2	0.000
Expected decrease in income	58.0 (57.9–58.0)	15.8 (15.8–15.8)	−42.2	−2,224	0.000
Expected increase in debt	49.1 (49.0–49.1)	14.0 (13.9–14.0)	−35.1	−1,877	0.000
Expectation of difficulty getting food and basis supplies	30.9 (30.8–30.9)	20.1 (20.0–20.1)	−10.8	−571.5	0.000

Likewise, 26% of the participants mentioned having been diagnosed with a mental disorder during their lives, while 15.2% reported having accessed mental health treatment during the last 12 months in W1. Around 34% reported having hypertension, obesity, and/or diabetes.

People who reported feeling lonely or isolated increased significantly between waves (15.2–19.6%, *p* < 0.05). In addition, there was a significant increase in the perceived lack of space in the home over time (12.8–21.7%, *p* < 0.01).

Between Waves 1 and 2 there was a large and significant reduction in economic uncertainty, both in the expectation of decreased household income (58.0–15.8%, *p* < 0.01) and the expectation of increased household debt (49.1–14.0%, *p* < 0.01). There is also a significant reduction in the expectation of facing difficulties in obtaining food and basic supplies (30.9–20.1%, *p* < 0.01).

As seen in Table 3, there is a significant difference in the distribution of psychological distress by sex, age, and wave. In both waves, moderate to severe anxiety-depression symptoms are more prevalent in women than in men (30.5 vs. 14.7% in W1, *p* < 0.001; 33.2 vs. 20.7% in W2, *p* < 0.001). There was a significant increase in psychological distress in all groups (*p* < 0.001). The largest increase was observed for men (14.7–20.7%) and younger participants (21.1–27.7%).

**Table 3:** Psychological distress by sex, age and wave.

				**Age group**	
	**Male**	**Female**	**18–35**	**36–59**	**60+**
Psychological distress W1 %	14.7	30.5	21.1	22.7	24.8
(95% CI)	(14.6–14.7)	(30.5–30.5)	(21.1–21.1)	(22.7–22.8)	(24.7–24.9)
Psychological distress W2 %	20.7	33.2	27.7	27.2	25.3
(95% CI)	(20.7–20.8)	(33.2–33.3)	(27.7–27.7)	(27.2–27.3)	(25.2–25.4)

As shown in Table 4, the results of the linear regression models using the data panel showed that being female, living in central Chile, living in overcrowded households (fewer bedrooms per person), having a perceived lack of space, loneliness, and having received mental health treatment within the past year are significantly associated with psychological distress during the pandemic (*p* < 0.05). Younger age is significantly associated with higher levels of depressive symptoms.

**Table 4:** Regression models (W1, W2 and panel data).

**Variable**	**W1 anxiety**	**W2 anxiety**	**Panel**	**W1**	**W2**	**Panel**	**W1**	**W2**	**Panel#**
			**anxiety**	**depression**	**depression**	**depression**	**Psy distress**	**Psy distress**	**Psy distress**
Loneliness	0.345^**^	0.605^**^	0.486^**^	0.614^**^	0.370^**^	0.501^**^	0.958^**^	0.974^**^	0.987^**^
	(0.121)	(0.102)	(0.0842)	(0.114)	(0.102)	(0.0872)	(0.174)	(0.151)	(0.126)
Female	0.376	0.331	0.364^**^	0.273	0.412	0.371^*^	0.649	0.741^*^	0.733^**^
	(0.197)	(0.169)	(0.137)	(0.201)	(0.212)	(0.154)	(0.358)	(0.295)	(0.246)
Center	0.337	0.269	0.230	0.253	0.202	0.249	0.593	0.473	0.481^*^
	(0.191)	(0.174)	(0.138)	(0.162)	(0.210)	(0.134)	(0.316)	(0.321)	(0.236)
South	0.101	0.188	0.0944	0.203	0.0191	0.162	0.306	0.207	0.257
	(0.193)	(0.187)	(0.137)	(0.164)	(0.210)	(0.131)	(0.316)	(0.344)	(0.237)
Age	0.00900	−0.00773	0.00242	−0.0146^*^	−0.0143	−0.0137^*^	−0.00555	−0.0221^*^	−0.0113
	(0.00620)	(0.00644)	(0.00482)	(0.00694)	(0.00806)	(0.00565)	(0.0115)	(0.0103)	(0.00801)
Rooms per person	−0.446^**^	−0.180	−0.293^**^	−0.156	−0.157	−0.0995	−0.602^**^	−0.337	−0.393^**^
	(0.143)	(0.138)	(0.104)	(0.108)	(0.146)	(0.0882)	(0.200)	(0.235)	(0.149)
Children<10 years old	0.0805	−0.0668	0.0167	0.116	−0.419	−0.107	0.199	−0.487	−0.0905
	(0.234)	(0.186)	(0.157)	(0.232)	(0.239)	(0.174)	(0.410)	(0.342)	(0.282)
Ln income	−0.258	−0.0264	−0.128	−0.225	−0.152	−0.151	−0.482	−0.181	−0.280
	(0.173)	(0.112)	(0.106)	(0.154)	(0.128)	(0.103)	(0.305)	(0.185)	(0.184)
Expectation difficulty getting food	−0.0745	0.395	0.154	−0.111	0.0278	−0.0544	−0.182	0.421	0.101
	(0.222)	(0.247)	(0.176)	(0.204)	(0.208)	(0.160)	(0.391)	(0.402)	(0.301)
Lack of space in the home	0.115	0.694^**^	0.561^**^	0.167	0.587^*^	0.498^*^	0.280	1.280^**^	1.057^**^
	(0.298)	(0.237)	(0.202)	(0.228)	(0.274)	(0.202)	(0.467)	(0.453)	(0.359)
Income reduction expectation	−0.452	0.690^**^	−0.00712	−0.201	0.657^**^	0.139	−0.651	1.347^**^	0.133
	(0.231)	(0.262)	(0.191)	(0.208)	(0.224)	(0.165)	(0.391)	(0.431)	(0.313)
Expectation of increasing debt	0.342	−0.103	0.241	0.0960	0.506	0.238	0.437	0.403	0.477
	(0.221)	(0.306)	(0.194)	(0.190)	(0.312)	(0.177)	(0.338)	(0.564)	(0.313)
Physical health status	0.0653	0.175	0.108	−0.0120	0.285	0.154	0.0554	0.459	0.263
	(0.193)	(0.191)	(0.142)	(0.179)	(0.217)	(0.153)	(0.320)	(0.325)	(0.240)
Mental health diagnosis	0.521^*^	0.283	0.342	−0.0221	0.145	0.0484	0.502	0.426	0.392
	(0.256)	(0.231)	(0.186)	(0.206)	(0.298)	(0.212)	(0.406)	(0.477)	(0.357)
Mental health treatment	0.419	0.598^*^	0.533^*^	−0.0683	0.790	0.416	0.348	1.388^*^	0.947^*^
	(0.348)	(0.286)	(0.237)	(0.341)	(0.423)	(0.300)	(0.608)	(0.542)	(0.437)
High school or lower	−0.0227	0.194	0.0825	0.153	0.442	0.336	0.129	0.634	0.416
	(0.327)	(0.287)	(0.232)	(0.238)	(0.259)	(0.181)	(0.501)	(0.463)	(0.356)
Higher education	0.0711	0.158	0.114	0.248	0.642^*^	0.436^*^	0.319	0.801	0.549
	(0.355)	(0.314)	(0.250)	(0.275)	(0.308)	(0.221)	(0.567)	(0.501)	(0.393)
Wave			−0.00520			0.524^**^			0.521
			(0.161)			(0.174)			(0.292)
Constant	4.591	1.301	2.659	4.507^*^	3.335	2.912	9.075^*^	4.676	5.580^*^
	(2.474)	(1.586)	(1.540)	(2.204)	(1.841)	(1.503)	(4.325)	(2.628)	(2.675)
Observations	704	663	1,367	705	661	1,366	703	661	1,364
R-squared	0.166	0.322	0.199	0.192	0.277	0.214	0.183	0.368	0.246

*Robust standard errors in parentheses*.

** *p < 0.01*,

* *p < 0.05*.

# *After Bonferroni correction in the main regression model, the variables loneliness, female and lack of space in the home remain significant (p < 0.1). The variable rooms per person is at the limit of significance (p = 0.153). The variables center and mental health treatment are no longer significant*.

It should also be noted that income, physical health status, and the presence of children under 10 in the household were not significantly associated with psychological distress.

Regarding the specific models according to the study wave, expecting reduced household income was significantly associated with psychological distress in Wave 2 (*p* < 0.01), while having been previously diagnosed with a mental health condition was significantly associated with anxious symptoms in Wave 1 (*p* < 0.05).

Since regression models revealed differences by sex and age, we generate alternative models stratified by sex and age category from the panel data (see Supplementary Tables 1, 2). Overall, gender-stratified models (Supplementary Table 1) show that loneliness, perceived lack of household space, and having accessed mental health treatment in the past year are significantly associated with psychological distress in both men and women. However, for women, overcrowded household conditions and a history of diagnosed mental health problems are significantly associated with higher levels of psychological distress, while for men, living in central Chile and younger age are significant. Interestingly, for men, the association between psychological distress and a history of a mental health diagnosis is reversed: men who have been diagnosed have lower levels of psychological distress.

The results of alternative regression models differentiated by age (Supplementary Table 2) show that loneliness significantly affects all age groups in terms of increased psychological distress. In addition, women aged 36–59 years-old and young people under 35 years-old appear to be particularly affected by a higher burden of anxious and depressive symptoms during the pandemic. Overcrowded household conditions mainly affect people aged 36–59. Finally, people aged 18–35 years with physical health problems (hypertension, obesity, or diabetes) and a history of mental health treatment in the past year have significantly higher levels of psychological distress during the pandemic.

## Discussion

To our knowledge, this is the first longitudinal survey of psychological distress based on a nationally representative sample during the COVID-19 outbreak in Chile. Our primary aim was to assess the levels of psychological distress at two time points during the pandemic. Overall, our sample exhibited relatively high levels of psychological distress, with ~22.6 and 27.0% of participants reporting moderate to severe anxiety and depressive symptoms in W1 and W2, respectively. This suggests a significant increase in psychological distress between Waves 1 and 2 (4.4%).

Previous studies have showed a non-significant change or even a decrease in anxious and depressive symptoms since the start of the pandemic in community samples (7–11, 13, 32). This suggests that anxious and depressive symptoms are sensitive to specific sociocultural, economic, and political conditions, and changes in the prevalence of mental health problems depend on the extent of local effects from the pandemic. The differences in psychological distress may also be related to social welfare and health care systems and governments' specific responses to the crisis.

Our results show a slightly lower burden of psychological distress than reported by international studies conducted during the early phases of the COVID-19 pandemic, which show that approximately one third of community samples have anxious or depressive symptoms (1, 2). This comparison should be treated with caution, as these studies use different scales and were conducted over different time periods. We can also compare our results with those from a pre-pandemic longitudinal survey in Chile (ELSOC, https://coes.cl/encuesta-panel/). That study shows that between 2016 and 2018 the average prevalence of depressive symptoms (PHQ-2 ≥ 3) was 20.6%. Meanwhile our prevalence was 25.4% in W1 and 32.2% in W2, which could suggest a significant increase in depressive symptoms due to the pandemic. Note that while the ELSOC sample shares some characteristics with our data set, the pre-pandemic estimates do come from a different community sample.

Our secondary aim was to identify groups that are more vulnerable to psychological distress during the pandemic. Overall, the results of this study show that being female, living in central Chile, or in overcrowded households, having a perceived lack of space in the home, loneliness, and having received mental health treatment within the last year are significantly associated with psychological distress during the COVID-19 pandemic.

Our findings suggest that living in central Chile increases the risk of psychological distress. This may be due to the fact that this area is primarily urban and include the capital, Santiago, which had 70% of the COVID-19 cases by September 2020 with a proportional number of deaths (21, 33). This region also had the most restrictive mobility restrictions with the longest lockdown. Unsurprisingly, other studies have showed that people living in urban areas with the strictest confinement measures and the highest COVID-19 infection and mortality rates report poorer mental health indicators (1, 15, 17).

It is well-known that women are more often exposed to social disadvantages, they are more likely than men to have informal employment contracts (34), and they suffer more frequently from anxious and depressive symptoms (35). Women's vulnerability could be exacerbated in periods of economic instability such as the current one (36, 37). In Chile, as in other Latin American countries, women tend to perform many roles simultaneously (employees, housewives, and caregivers) and were more likely to experience additional burdens before and then during the pandemic (22, 38). This helps to explain why women aged 36–59 are particularly affected by the pandemic in terms of mental health.

Living in overcrowded households and having a perceived lack of space in the home are significantly associated with psychological distress. It is interesting that the effect of living in overcrowded households was observed in the first wave, while the perception of lack of space and its effect on psychological distress was greater in the second wave, perhaps because people have had more experience living in confined conditions.

Household overcrowding is a major public policy issue in Chile. The combination of rapid urbanization and a housing market organized around profit maximization with weak regulation led to the construction of small dwellings characterized by overcrowding (23). A previous study conducted in Chile also found that increased overcrowding is associated with more depressive symptoms (39). International studies conducted during the COVID-19 pandemic found a significant association between living in poor quality housing conditions and depressive symptoms (16, 40, 41).

The physical characteristics of the household and the perception of lack of space during quarantine affecting people's mood could be associated with lack of privacy or poor conditions for people's daily activities. It should be noted that our results also show that overcrowding significantly affects women aged 36–59, which is consistent with previous studies (42). In pandemic conditions, women are more likely to deal with the simultaneous burden of work and childcare, which can be even more difficult in overcrowded households. However, in contrast to other studies (1, 3), ours show that the presence of children in the household was not significantly associated with psychological distress.

One of our main findings is a significant association between loneliness and psychological distress. Like other studies (32, 43), our results also show that, despite relaxed lockdown measures over time, the proportion of people who reported feeling lonely or isolated increased significantly between W1 and W2, suggesting that this may be associated with reduced social interactions during the pandemic.

Previous studies have suggested that physical distancing and lockdown measures could be associated with a sense of loneliness and social isolation, and thus affect psychological well-being (32, 43). There is convincing longitudinal evidence suggesting that loneliness precedes anxiety and depressive symptoms (44, 45). Loneliness is being defined as subjective distress resulting from a discrepancy between desired and perceived social connectedness, while social isolation is an objective deficit in the number of relationships and frequency of social contact (45). This allows us to understand the possible co-presence of household overcrowding and loneliness, since the feeling of connectedness can be affected despite the physical presence of others.

Our study shows that people with previous mental health diagnoses were not more likely to experience psychological distress over time, although they were more likely to experience anxiety symptoms during the first months of the COVID-19 outbreak. The high uncertainty caused by the onset of the pandemic likely caused excessive worry about future events, which is a key component of anxiety (46). However, it is not possible for us to conclude that the current pandemic is more stressful for people with a history of mental disorders.

The emerging literature on COVID-19 provides insight for our findings. A British study showed that adults with pre-existing mental illness diagnoses experienced higher levels of anxious and depressive symptoms during the first weeks of quarantine; but found little difference overall mental health outcomes based on previous psychiatric issues (11). A study conducted in the Netherlands shows that people with depressive and anxiety disorders scored higher on all symptom scales than did individuals without these disorders; however, people without these disorders showed a greater increase in symptoms during the pandemic (47).

In contrast, our findings show that people who have received mental health treatment within the last year showed higher levels of psychological distress. People who have recently received or are currently undergoing psychological treatment tend to be more sensitive to stressors compared with the general population (48). For these people, travel restrictions, fear of infection, and changes in the organization of health-care services could interrupt access to treatment, impacting on their mental health (49).

Our results are consistent with other COVID-19 studies showing higher psychological distress, especially depressive symptoms, among people under 35 years old (1, 50). Several studies have suggested that younger people may be particularly affected by social contact deprivation, since they are in a period of life characterized by a greater need for peer interaction (2, 13). In addition, this group includes higher education students who may experience higher levels of depressive symptoms due to the closure of education institutions and difficulties in distance learning (50). Our results indicate that this may particularly affect young men.

Our results also show that economic uncertainty significantly diminished between W1 and W2. While the expectation of decreased household income has a significant effect on psychological distress in W2, the expectation of increased household debt has no significant effect in either wave. A previous study on COVID-19 and psychological distress in Chile showed that almost half of the participants thought that they would face reduced income due to having to stop working as a direct or indirect effect of the pandemic, and that this was significantly associated with psychological distress (26). The expectation of declining income may contribute to triggering a sense of hopelessness, which represents a central aspect of depressive feelings.

In relation to debt and access to basic supplies, our findings may be related to the effects of government social policies. To alleviate rising unemployment and the impact of COVID-19 pandemic, the Chilean government launched a food supply campaign in May 2020, and announced cash transfers targeted at the most affected households. In addition, at the end of July, the National Congress passed a law allowing workers to withdraw up to 10% of their pension funds. By November 2020, 10.1 million people had requested the withdrawal, which is 92% of everyone in the pension system (51). In our sample, 69.4% reported having withdrawn a portion of their pension funds in W2 (unreported result). According to preliminary data, a large proportion of households used these resources to pay off debts (52).

Overall, this study suggests that the impact of risk factors vary over time and population. Qualitative research is needed to understand the mechanisms through which the risk factors identified in this study are related to psychological distress in the context of COVID-19 pandemic, and to identify the coping strategies that people use or could use to adapt to this adverse context.

### Policy Implications

Our study has important implications for public health and social policy. In the current context, more attention and assistance should be given to vulnerable groups such as women, people ≤35 years old, those with weak social networks and/or living in overcrowded conditions, and those who were already receiving mental health treatment. Addressing loneliness, improving mental health care access, and addressing housing issues may be important in reducing anxiety and depressive symptoms (40, 44, 53), especially for women.

Historically interventions to reduce loneliness have focused on in-person socializing (45). Thus, the development of interventions in the context of physical distancing measures and quarantines represents a major challenge for social policy. Digital methods of communication have proven beneficial when physical interaction is not possible (53, 54).

Several health systems have adopted digital mental health as a tool to address the treatment gap that the COVID-19 pandemic has widened (49). Given that mental health services are limited and delayed because of extended quarantine periods and redeployment of health care resources to COVID-19 (49), remote services such as internet-based interventions or telephone hotlines, are important tools for providing preventive care and treatment (55).

This study also suggests the need for an interdisciplinary approach informing housing policies. This is a relevant perspective when considering future “stay-at-home” scenarios. However, there is a lack of evidence on housing interventions that are shown to have a positive effect on people's health (56, 57). Future research is thus needed.

### Strengths and Limitations

One of the main strengths of this study is that it was based on a nationally representative longitudinal dataset in a developing country. Although this study lacks a pre-pandemic baseline, it still shows changes in participants' mental health during the pandemic over time. This study has also a wide heterogeneity and good stratification in multiple socio-demographic groups.

However, findings should be interpreted in the context of certain limitations. An important limitation is the data collection strategy used (telephone survey). In addition, we have used an ultra-brief self-report scale to measure anxious and depressive symptoms (PHQ-4). This may have resulted in overestimating prevalence.

Another limitation is that we abbreviated the three-item loneliness scale into a single combined item, treating loneliness and perceived social isolation as a uni-dimensional construct. Future studies should use full versions of the loneliness scale.

## Conclusion

This study showed a significant increase in the prevalence of psychological distress during the COVID-19 outbreak in Chile. We have identified certain groups who were more vulnerable to psychological distress: woman, those living in central Chile (Metropolitan Region), those living in overcrowding households and having a perceived lack of space, feeling lonely and isolated, and having received mental health treatment within the last year.

These findings highlight the need for public health and social policies addressing individual needs and social determinants of mental health, especially social connection disruptions and quarantine-related stressors. Given that recovery from the pandemic will take several years, and that high levels of psychological distress in the population may create a burden for social and economic recovery, this study can help suggest types of social policies needed to deal with pandemic-related difficulties and long-term consequences.

Further longitudinal research is required, preferably using mixed methods and an interdisciplinary approach, to assess the impact of the COVID-19 pandemic on mental health across different societal groups.

## Data Availability Statement

The raw data supporting the conclusions of this article will be made available by the authors, without undue reservation.

## Ethics Statement

Ethical approval was obtained from the Ethics Committee of the Faculty of Economics and Business of the Universidad de Chile. Because the survey was conducted by telephone, the consent form was read by the interviewers and participants gave their verbal consent before responding, which was audio recorded. The ethics committee approved this verbal consent procedure.

## Author Contributions

FD and ÁJ-M were involved in the design of the study and participated equally in the writing of the manuscript. FD analyzed the data. Both authors approved the final manuscript.

## Funding

FD and ÁJ-M received funding from ANID/Millennium Science Initiative, grant Millennium Nucleus in Social Development (DESOC), NCS17_015. ÁJ-M also received funding from ANID/Millennium Science Initiative Program, grant NCS2021_81 and ICS13_005, and ANID/FONDECYT POSTDOCTORADO/2020-3200944. The funders had no role in study design, data collection and analysis, interpretation of data and in the preparation of the manuscript.

## Conflict of Interest

The authors declare that the research was conducted in the absence of any commercial or financial relationships that could be construed as a potential conflict of interest.

## Publisher's Note

All claims expressed in this article are solely those of the authors and do not necessarily represent those of their affiliated organizations, or those of the publisher, the editors and the reviewers. Any product that may be evaluated in this article, or claim that may be made by its manufacturer, is not guaranteed or endorsed by the publisher.
