# Supplementary material for: A Longitudinal Nationwide Study of Psychological Distress During the COVID-19 Pandemic in Chile
Source: Front Psychiatry. 2022 Feb 24;13:744204. doi: 10.3389/fpsyt.2022.744204 (PMC8907540; doi:10.3389/fpsyt.2022.744204)
Supplement: Supplementary file 1 [file Data_Sheet_1.docx]

Supplementary Material

|  |  |  |  |  |  |
| --- | --- | --- | --- | --- | --- |

**Supplementary Table 1 | Regression models by sex**

|  |  | |
| --- | --- | --- |
|  | **Psychological distress** | |
| **Variables** | **Female Panel** | **Male Panel** |
|  |  |  |
| Loneliness | 0.813**  (0.166) | 1.133**  (0.190) |
|  |  |  |
| Center | 0.170  (0.324) | 0.631*  (0.313) |
|  |  |  |
| South | -0.178  (0.332) | 0.354  (0.350) |
|  |  |  |
| Age | 0.00384  (0.0107) | -0.0237*  (0.0105) |
|  |  |  |
| Rooms per person | -0.720**  (0.208) | -0.0408  (0.201) |
|  |  |  |
| Children<10 years old | -0.192  (0.310) | -0.0720  (0.410) |
|  |  |  |
| Ln income | 0.161  (0.215) | -0.435  (0.265) |
|  |  |  |
| Expectation difficulty getting food | -0.0619  (0.331) | 0.282  (0.455) |
| Lack of space in the home | 1.004*  (0.427) | 1.078*  (0.471) |
| Income reduction expectation | 0.255  (0.319) | -0.0347  (0.470) |
| Expectation of increasing debt | 0.401  (0.342) | 0.474  (0.459) |
| Physical health status | 0.258  (0.280) | 0.196  (0.350) |
|  |  |  |
| Mental health diagnosis | 1.581**  (0.362) | -1.182**  (0.426) |
|  |  |  |
| Mental health treatment | 0.988*  (0.475) | 1.407*  (0.596) |
|  |  |  |
| High school or lower | 0.0343  (0.466) | 0.805  (0.524) |
|  |  |  |
| Higher education | -0.105  (0.520) | 0.925  (0.537) |
|  |  |  |
| Wave | 0.330  (0.362) | 0.500  (0.366) |
|  |  |  |
| Constant | 0.844  (2.969) | 7.608*  (3.873) |
|  |  |  |
|  |  |  |
| Observations | 713 | 651 |
| R-squared | 0.264 | 0.305 |
| Robust standard errors in parentheses | | |
| * p<0.05, ** p<0.01 |  |  |

**Supplementary Table 2 | Regression models by age**

|  |  | | |
| --- | --- | --- | --- |
|  | **Psychological distress** | | |
| **Variables** | **18-35 Panel** | **36-59 Panel** | **60+ Panel** |
|  |  |  |  |
| Loneliness | 0.904** | 1.268** | 0.626** |
|  | (0.180) | (0.195) | (0.235) |
| Female | -0.182 | 1.543** | 0.251 |
|  | (0.408) | (0.320) | (0.476) |
| Center | 0.116 | 0.784* | 0.944* |
|  | (0.417) | (0.351) | (0.453) |
| South | 0.647 | 0.492 | 0.614 |
|  | (0.465) | (0.380) | (0.471) |
| Age | -0.0950** | 0.00502 | -0.0745 |
|  | (0.0249) | (0.0258) | (0.0401) |
| Rooms per person | -0.116 | -0.972** | -0.514 |
|  | (0.237) | (0.292) | (0.299) |
| Children<10 years old | 0.104 | 0.250 | -0.907 |
|  | (0.445) | (0.351) | (0.541) |
| Ln income | -0.526 | 0.0371 | -0.345 |
|  | (0.277) | (0.236) | (0.378) |
| Expectation difficulty getting food | -0.0523 | -0.155 | 0.356 |
|  | (0.499) | (0.350) | (0.499) |
| Lack of space in the home | 0.759 | 0.761 | 1.747 |
|  | (0.414) | (0.500) | (0.903) |
| Income reduction expectation | 0.0859 | 0.342 | 0.505 |
|  | (0.470) | (0.373) | (0.672) |
| Expectation of increasing debt | -0.270 | 0.549 | 0.821 |
|  | (0.432) | (0.381) | (0.645) |
| Physical health status | 0.985** | 0.206 | -0.0548 |
|  | (0.374) | (0.294) | (0.446) |
| Mental health diagnosis | 0.122 | 0.894 | 0.305 |
|  | (0.384) | (0.500) | (0.500) |
| Mental health treatment | 2.498** | 0.503 | 1.138 |
|  | (0.689) | (0.584) | (0.673) |
| High school or lower | 0.729 | 0.253 | 0.989 |
|  | (1.365) | (0.481) | (0.561) |
| Higher education | 1.757 | -0.347 | 1.142 |
|  | (1.389) | (0.531) | (0.654) |
| Wave | 0.536 | 0.476 | 0.0329 |
|  | (0.477) | (0.343) | (0.560) |
| Constant | 10.60* | 0.465 | 11.05 |
|  | (4.269) | (3.794) | (6.104) |
|  |  |  |  |
| Observations | 374 | 621 | 369 |
| R-squared | 0.336 | 0.352 | 0.237 |
| Robust standard errors in parentheses | | |  |
| * p<0.05, ** p<0.01 |  |  |  |

**
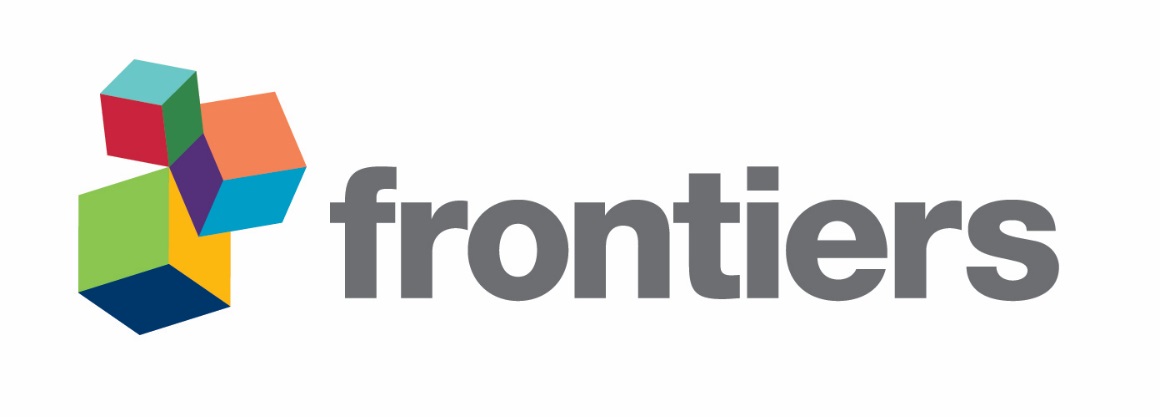
**
